# The specific seroreactivity to ∆Np73 isoforms shows higher diagnostic ability in colorectal cancer patients than the canonical p73 protein

**DOI:** 10.1038/s41598-019-49960-x

**Published:** 2019-09-19

**Authors:** María Garranzo-Asensio, Ana Guzmán-Aránguez, Carmen Povés, María Jesús Fernández-Aceñero, Ana Montero-Calle, María Ángeles Ceron, Servando Fernandez-Diez, Nuria Rodríguez, Marta Gómez de Cedrón, Ana Ramírez de Molina, Gemma Domínguez, Rodrigo Barderas

**Affiliations:** 1 Departamento de Bioquímica y Biología Molecular, Facultad de Óptica y Optometría, Universidad Complutense de Madrid, E-28040 Madrid, Spain; 2 UFIEC, Chronic Disease Programme, Instituto de Salud Carlos III, Majadahonda, E-28220 Madrid Spain; 3 Gastroenterology Unit, Hospital Universitario Clínico San Carlos, E-28040 Madrid, Spain; 4 Surgical Pathology Department, Hospital Universitario Clínico San Carlos, E-28040 Madrid, Spain; 5 Medical Oncology Department, Hospital Universitario La Paz, E-28046 Madrid, Spain; 6 Molecular Oncology and Nutritional Genomics of Cancer, IMDEA-FOOD, E-28049 Madrid, Spain; 7 Departamento de Medicina, Facultad de Medicina, Instituto de Investigaciones Biomédicas “Alberto Sols”, CSIC-UAM, E-28029 Madrid, Spain

**Keywords:** Tumour biomarkers, Diagnostic markers, Tumour biomarkers

## Abstract

The p53-family is tightly regulated at transcriptional level. Due to alternative splicing, up to 40 different theoretical proteoforms have been described for p73 and at least 20 and 10 for p53 and p63, respectively. However, only the canonical proteins have been evaluated as autoantibody targets in cancer patients for diagnosis. In this study, we have cloned and expressed *in vitro* the most upregulated proteoforms of p73, ΔNp73α and ΔNp73β, for the analysis of their seroreactivity by a developed luminescence based immunoassay test using 145 individual plasma from colorectal cancer, premalignant individuals and healthy controls. ∆Np73α seroreactivity showed the highest diagnostic ability to discriminate between groups. The combination of ∆Np73α, ∆Np73β and p73 proteoforms seroreactivity were able to improve their individual diagnostic ability. Competitive inhibition experiments further demonstrated the presence of unique specific epitopes in ΔNp73 isoforms not present in p73, with several colorectal patients showing unique and specific seroreactivity to the ΔNp73 proteoforms. Overall, we have increased the complexity of the humoral immune response to the p53-family in cancer patients, showing that the proteoforms derived from the alternative splicing of p73 possess a higher diagnostic ability than the canonical protein, which might be extensive for p53 and p63 proteins.

## Introduction

The p53 family of genes includes p53, p63 and p73. All three p53-family proteins have a very similar domain organization, are expressed in a similar set of alternative isoforms, and are subjected to similar post-translational modifications. However, important differences in their biological role have been revealed, showing that p53-family paralogs have acquired a high degree of functional specificity since their duplication and divergence during evolution^1–3^.

p53 is a powerful tumor suppressor, as proven by a profusion of *in vivo* models, which is frequently mutated in human cancers^1,4,5^. In addition, a wealth of data shows that p63 and p73 have a role in tumor suppression. Studies with p63^+/−^ and p73^+/−^ heterozygous mice revealed a consistent connection with cancer. p63^+/−^ and p73^+/−^ mice develop spontaneous tumors and show a median survival time a few months longer than that of p53^+/−^ mice^6^. A number of studies have shown that TAp73 and TAp63 can induce cell-cycle arrest, senescence, DNA repair, and apoptosis in response to chemotherapeutic drugs, independently of p53^7–9^. In addition, despite p63 and p73 being barely mutated in cancer, they are aberrantly expressed in cancer. In particular, ΔN isoforms of p63 and p73 are frequently overexpressed in a wide range of tumors, where they are associated with poorer prognosis^10^. Moreover, forced expression of ΔNp73 promotes transformation in experimental models^11,12^. Thus, upregulation of ΔNp63 or ΔNp73 isoforms may be a common mechanism to inactivate the respective TA isoforms during tumorigenesis.

p53 autoantibodies are reported in many cancer patients^13,14^. Indeed, p53 is considered as the main cancer autoantigen that should be included in any blood-based cancer diagnostic test because of its specificity for detecting cancer^15–17^. Moreover, p53 autoantibodies reevaluation in sera of cancer patients has shown a growing interest because of their role in early cancer detection^17^. Beyond the presence of autoantibodies to p53, and despite the presence of autoantibodies to p63 and p73 in the sera of cancer patients’ non-seroreactive to p53, the study of the roles of p63 and p73 autoantibodies in cancer have been almost dismissed^18–22^. In addition, no report has been focused on the analysis of the seroreactivity of the different proteoforms of the p53-family in cancer patients. This is probably because of the numerous protein isoforms encoded by each of these genes.

The three proteins share a common structure consisting of an N-terminal transactivation domain, a central highly conserved DNA binding domain and a C-terminal oligomerization domain. However, the three members of the TP53-family encode for multiple isoforms containing different protein domains due to alternative splicing from P1 and P2 promoters (one intragenic) with one more -P3- detected *in silico*, and alternative initiation of translation^23,24^. Despite its extensive regulation where different isoforms have a different N- or C-terminal, and different intraprotein amino acid sequences derived from the alternative spliced exons, only the canonical reference proteins p53, p63 and p73 -or point mutated p53- have been tested for autoantibody screening and cancer detection^14,25^.

**Figure 1:**
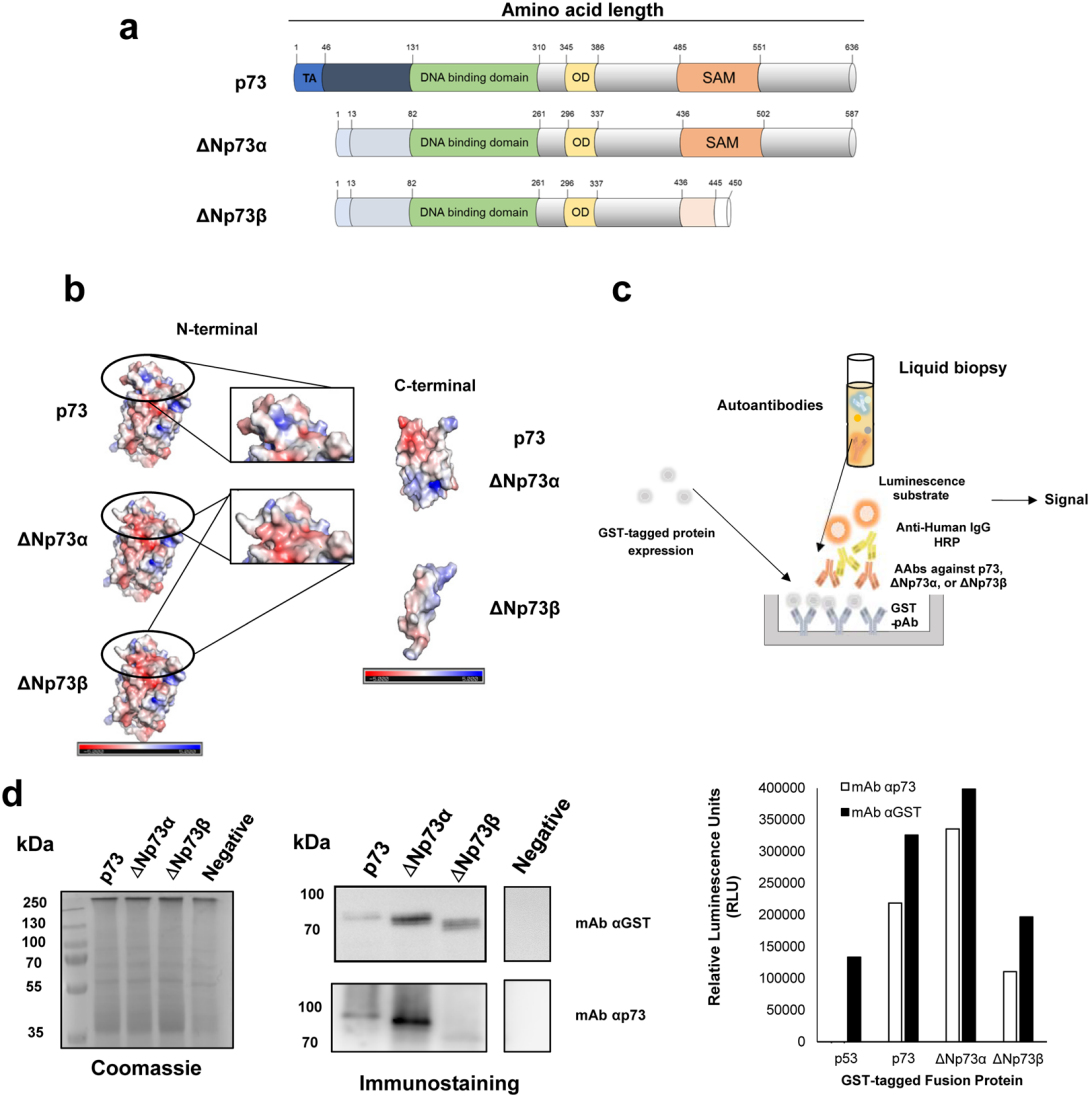
Scheme of the developed approach to evaluate the humoral immune response to p73 and ∆Np73 isoforms. (**a**) The selected p73 isoforms for the study differ from the canonical sequence on their N-terminal end (∆Np73α), or in the N- and C-terminal ends (∆Np73β). TA, transactivation domain; OD, oligomerization domain. (**b**) Prediction of the 3D structure of indicated p73 proteoforms and their predicted electrostatic potential. Electropositive and electronegative charged regions are colored in blue and red, respectively. Neutral regions are colored in white. (**c**) For the evaluation of autoantibody levels against them, ELISA plates were coated with GST pAb to capture GST-tagged p73-family proteins. After incubation with plasma samples containing the autoantibodies followed by an anti-Human IgG-HRP antibody, a luminescence substrate is added to obtain a quantifiable signal. (**d**) IVTT protein expression was verified by immunostaining, and ELISA using antibodies directed against p73 or the GST-tag. For cropped WB images, expressed proteins were run in the same gel under same experimental conditions as the negative controls and processed in parallel.

A major step for the development of immunoassays consists of the production of proteins with good yield, purity, stable and integral for functional protein studies. Here, we have just-in-time produced the canonical p73 protein and two aberrantly overexpressed ∆Np73 isoforms, frequently upregulated in cancer, using a cell-free system for its direct use to determine the presence of specific autoantibodies against them (Fig. 1). This system is highly efficient, and has succeeded at producing thousands of different proteins for the identification of disease-specific antibodies and host-pathogen interaction profiling^26–28^. Our results, achieved using 145 plasma samples from colorectal cancer (CRC) patients, patients carrying premalignant colorectal lesions, and controls, demonstrated that ΔNp73 showed a specific seroreactivity different from that of p73 with a higher diagnostic ability to discriminate between colorectal cancer patients, and controls, and especially premalignant individuals and controls which may have an important impact on cancer prevention to predict premalignant tumours.

## Results

The p53-family of proteins has been analyzed as target of specific autoantibodies in cancer patients using the canonical proteins, except for p53, whose seroreactivity has been also evaluated using the most frequent p53 point mutants found in cancer and different N-terminal and C-terminal deletions of the protein to identify masked epitopes of the cancer autoantigen^14^. However, even though the p53-family is composed of multiple proteoforms derived from two different promoters leading to different N- and/or C-terminal primary amino acid sequences, none of these proteoforms have been evaluated as potential autoantigens target of autoantibodies in cancer patients.

Therefore, due to the fact that these isoforms are highly overexpressed in cancer, we hypothesized that a differential seroreactivity to these unique “masked” epitopes could exist in the different isoforms. In addition, the fact that the different isoforms carry differential epitopes compared to the canonical proteins could contribute to increase the diagnostic ability of the target proteins, or even identify cancer patients with specific autoantibodies to any of the isoforms.

To address this question, we have evaluated the seroreactivity to three proteins of the p53-family fused to GST by an ELISA-based test using as model the canonical p73 protein and its ΔNp73α, and ΔNp73β isoforms^29^, which carry a different N-terminal and a different N- and C-terminal ends not present in the canonical p73 sequence, respectively (Fig. 1a,b). To further visualize the putative structural differences that would produce masked epitopes, 3D prediction models were obtained for the three proteins. 3D-models showed that the differences in the primary sequence produce important changes in the 3D-folding and in the electrostatic surface potential at both the N- and C-terminal end of the ∆Np73 proteoforms in comparison to p73 (Fig. 1b).

### *In vitro* protein expression of fusion proteins

For the development of our approach (Fig. 1c), we firstly transferred TP73, ΔNp73α, and ΔNp73β genes, and TP53 as control, from the donor vectors to a pANT7_cGST vector by means of LR clonase reactions and directly use the purified DNA for *in vitro* protein expression of the corresponding proteins fused to GST (Fig. 1d).

The success of protein expression was determined by probing the IVTT expression by WB and ELISA with an anti-GST monoclonal antibody that recognizes the GST tag in the C-terminal end of every fusion protein and an anti-p73 monoclonal antibody that specifically recognizes p73 and its ∆N proteoforms (Fig. 1d). 10 ng of GST fusion proteins were obtained per 1 µl of IVTT reaction, according to the GST control protein included in the assay as control (data not shown).

### ELISA-based test optimization for evaluation of the seroreactivity to p73 and its isoforms

**Figure 2:**
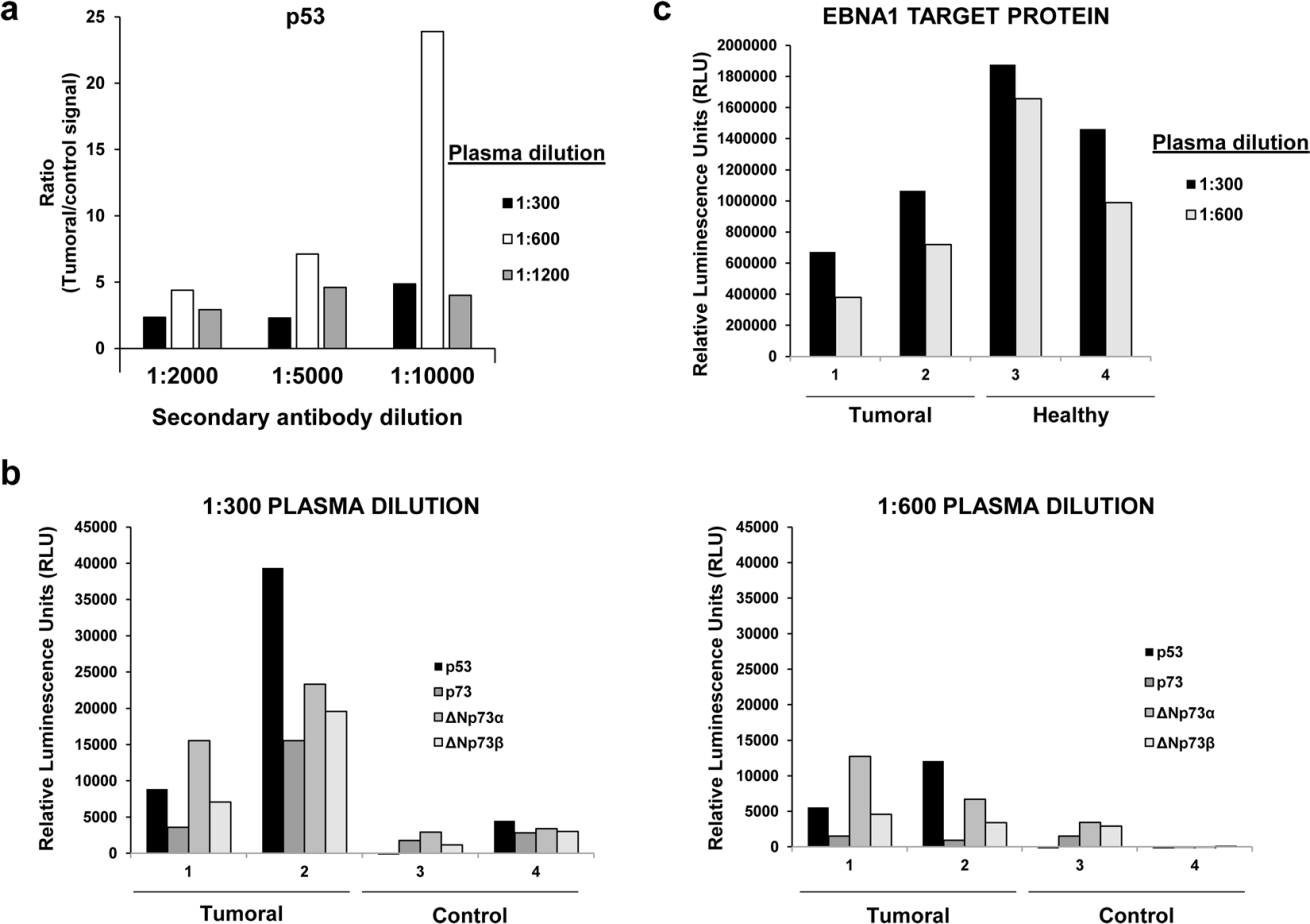
Optimization of the ELISA-based test for the evaluation of the seroreactivity to p73 and ΔNp73 isoforms. (**a**) Seroreactivity to p53, p73, ΔNp73α, and ΔNp73β of individual plasma samples either seroreactive or negative to p53 was assessed by ELISA at 1:300 and 1:600 dilutions with IVTT expressed proteins diluted with PBS and captured in 96-well ELISA plates. Signal ratios were calculated to determine the optimal conditions of the assay. (**b**) To assess the best dilution combination of plasma samples and secondary HRP-antibody, different secondary antibody dilutions were tested with p53 seroreactive (Tumoral plasma sample) and non-seroreactive (Non-tumoral plasma sample) samples diluted 1:300, or 1:600. (**c**) Seroreactivity to EBNA1 was measured as positive control of the assay.

We next used the fusion proteins to determine whether either ΔNp73α or ΔNp73β isoforms of p73 were able to induce a specific humoral immune response in cancer patients different from that observed for the canonical p73 protein. To this end, we optimized and validated the feasibility of the ELISA approach to determine the specific seroreactivity against the fusion proteins using plasma from cancer patients and controls (Fig. 2).

We firstly optimized the protein immobilization to the ELISA plates coated with anti-GST polyclonal antibody. We found an optimal IVTT reaction volume of 2.5 µl per ELISA well (data not shown). In a second step, we determined the optimal plasma and secondary antibody dilution for the detection of autoantibodies to p73 and its ∆Np73 isoforms by chemiluminescence (Fig. 2). We tested serial dilutions of a pool of CRC and control plasma samples known to be reactive to p53^15^ from 1:300 to 1:1200 to avoid the “hook” effect due to interferences caused by higher plasma concentrations^30^, and serial dilutions of secondary antibody from 1:2000 to 1:10000 (Fig. 2a). Better results were observed at a 1:300, and 1:600 dilutions of human plasma and 1:10000 dilution for the secondary antibody. Accordingly, we finally assessed with individual plasma which dilution would be optimal for further experiments (Fig. 2b), using EBNA1 as positive control of the seroreactivity for all individual plasma (Fig. 2c). In all cases, best results were obtained using a plasma dilution of 1:300. Interestingly, a significant specific differential seroreactivity to the ΔNp73 isoforms in comparison to the p73 canonical sequence, different from that observed for EBNA1, was observed (Fig. 2b,c).

### Evaluation of the diagnostic potential of the specific seroreactivity to p73, ΔNp73α and ΔNp73β proteoforms in colorectal cancer patients

Next, we proceeded to investigate their diagnostic effectiveness by the ELISA-based test using a total of 145 individual human plasma from CRC and premalignant patients, and controls and to determine whether a specific differential seroreactivity among them could exist. We used a total of 43 CRC plasma samples from patients at stages I-IV, 34 premalignant samples (low-grade or high-grade adenoma), and 68 healthy control plasma samples (Table 1).

**Table 1:** Clinical information of the colorectal cancer patients and controls used in the study.

Group		Sample size (N)	Overall age (mean ± SD)	Male (N)	Female (N)
Control	Asymptomatic	41	45 ± 7	12	29
	Negative colonoscopy	27	53 ± 10	10	17
Premalignant		34	59 ± 7	16	18
CRC	Stage I and II	14	76 ± 9	7	7
	Stage III	11	73 ± 10	8	3
	Stage IV	18	67 ± 12	6	12

**Figure 3:**
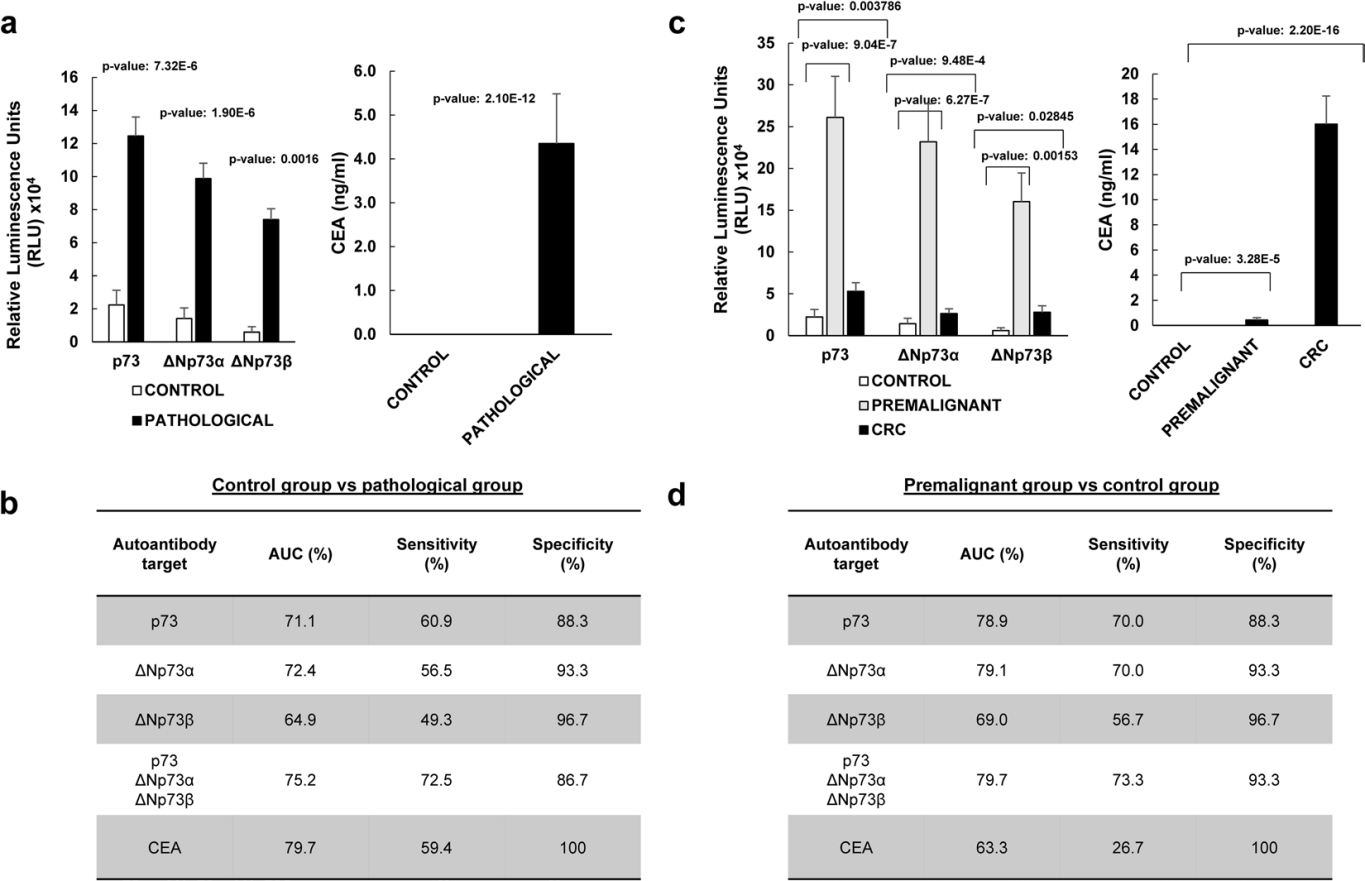
The analysis of the seroreactivity to p73, ΔNp73α, and ΔNp73β showed that ΔNp73α possesses a significant higher diagnostic ability than the other proteoforms to discriminate colorectal cancer patients, or premalignant individuals from healthy controls. (**a,b**) Analysis of the seroreactivity in comparison to CEA concentration in the pathological group (CRC patients and premalignant individuals) *vs* the control group. (**c,d**) Analysis of the seroreactivity in comparison to CEA concentration for indicated groups. (**a**) Statistical differences (*p*-value = 7.32e-06) were found comparing the mean value of the luminescence signal obtained for p73 in the control group (22299 ± 8909 RLU) and the pathological group –premalignant individuals with positive colonoscopy and CRC patients- (124577 ± 11469 RLU). When comparing seroreactivity to ΔNp73α, statistical differences (*p*-value = 1.90e-06) were also found between the control group (14155 ± 6426 RLU) and the pathological group (98858 ± 9167 RLU), as well as when comparing seroreactivity to ΔNp73β (*p*-value = 0.0016) between the control group (5889 ± 3264 RLU) and pathological subjects (73951 ± 6508 RLU). On the other hand CEA mean values were 0 for the control group and 4.35 ± 1.13 ng/ml for the pathological group (*p*-value = 2.1e-12). (**b**) Individual and combined ROC curves values for the seroreactivity to p73, ΔNp73α, or ΔNp73β were calculated to assess their predictive value for the pathological colorectal group, comparing healthy subjects with premalignant and CRC patients. Similar results were observed for the combined ROC curves of the seroreactivity to p73, ΔNp73α, and ΔNp73β than that of CEA. (**c**) Statistically significant differences were found comparing the seroreactivity and CEA among the three groups, being the seroreactivity differences between the control and the premalignant groups the higher regarding their autoantibody levels and their significance. Seroreactive mean values for p73 were 22299 ± 8909, 261054 ± 49056, and 52851 ± 10524; for ΔNp73α were 14155 ± 6426, 231933 ± 45282, and 26315 ± 5583, and for ΔNp73β were 5889 ± 3264, 160154 ± 34509, and 27796 ± 7821 the control, premalignant individuals and CRC groups, respectively. On the other hand, CEA mean values were 0, 0.44 ± 0.19, and 16.01 ± 2.23 ng/ml for the same groups. (**d**) Individual and combined ROC curves values for the seroreactivity to p73, ΔNp73α, or ΔNp73β were calculated to assess their predictive value for the detection of premalignant individuals from controls. Individual or combined seroreactive results to p73, ΔNp73α, and ΔNp73β to detect premalignant individuals were significantly higher than of CEA. All given values correspond to the trimmed mean ± SEM to compensate for extreme values. All measurements were taken in duplicate. *See* Supplementary Fig. S3 for all comparisons and data derived from ROC curves.

We firstly focused our analysis on the diagnostic effectiveness of the different constructs. A significant difference in the seroreactivity to all the p73-derived constructs was observed comparing the control plasmas, and the CRC and the premalignant individuals’ plasmas (Figs 3, S1, S2).

For the plasma samples from non-pathological control individuals, a mean value of 22299 ± 8909 RLU, 14155 ± 6426 RLU, and 5889 ± 3264 RLU, was achieved for p73, ∆Np73α, and ∆Np73β, respectively; while the colorectal pathological group reached a mean value at least 5 times higher 124577 ± 11469 RLU, 98858 ± 9167 RLU, and 73951 ± 3264 RLU (Fig. 3a).

Then, the diagnostic value of the fusion proteins was individually and collectively assessed using receiver operating characteristic (ROC) curves. p73, ΔNp73α, and ΔNp73β showed individually areas under the curve (AUCs) of 71.1%, 72.4%, and 64.9% respectively, with specificity and sensitivity up to 60.9% and 96.7% (Figs 3b, S3). In combination, they showed an AUC of 75.2% and a specificity and sensitivity of 86.7 and 72.5%, respectively, showing that the measurement of the ΔN proteoforms increases the diagnostic potential of the p73 canonical protein alone (Supplementary Fig. S3). More importantly, ΔNp73α showed a higher diagnostic ability than either p73 or ΔNp73β.

Interestingly, it was observed that the difference was mostly due to the autoantibody levels observed in the premalignant individuals (Supplementary Fig. S1). The premalignant individuals group reached a mean value at least 10 times higher than the control group, whereas CRC patients reached a value ranging between 2 and 5 times higher than the control group (Fig. 3c). Then, we proceed to determine whether the seroreactivity to p73, ∆Np73α, and ∆Np73β assessed by means of individual and combined ROC curves could predict the presence of premalignant colorectal lesions (Fig. 3d). p73, ΔNp73α, and ΔNp73β showed individually AUCs of 78.9%, 79.1%, and 69.0% respectively, with specificity and sensitivity up to 70% and 96.7% to discriminate premalignant individuals from controls. These values were considerably higher than that observed for CRC (Supplementary Fig. S3), with AUC values up to 67.4%. In combination, they showed an AUC of 79.7% and a specificity and sensitivity of 73.3 and 93.3%, respectively, to detect premalignant individuals. In contrast, for CRC their combined AUC was 61.5%, with a specificity and sensitivity of 90.0 and 59% (Supplementary Fig. S3).

Finally, we proceed to compare the seroreactivity results with the measurement in the same samples of the carcinoembryonic antigen (CEA) tumor marker. Importantly, whereas CEA values were able to discriminate CRC samples better than the humoral response to p73, ∆Np73α, and ∆Np73β individually or combined, and at similar levels the pathological group from the control group, results were significantly better for the humoral response to detect premalignant individuals (Figs 3 and S3).

Collectively, these results showed that ΔNp73α possess a higher diagnostic ability than either p73 or ΔNp73β, and especially to discriminate premalignant individuals from controls. More importantly, these results suggest the great potential of the measurement of the autoantibody responses to ΔNp73 proteoforms for the detection of premalignant colorectal lesions, which may have an important impact in clinics for cancer prevention to detect those individuals carrying premalignant colorectal tumors.

### Evaluation of ΔNp73 levels in tissue samples from CRC patients by qPCR

**Figure 4:**
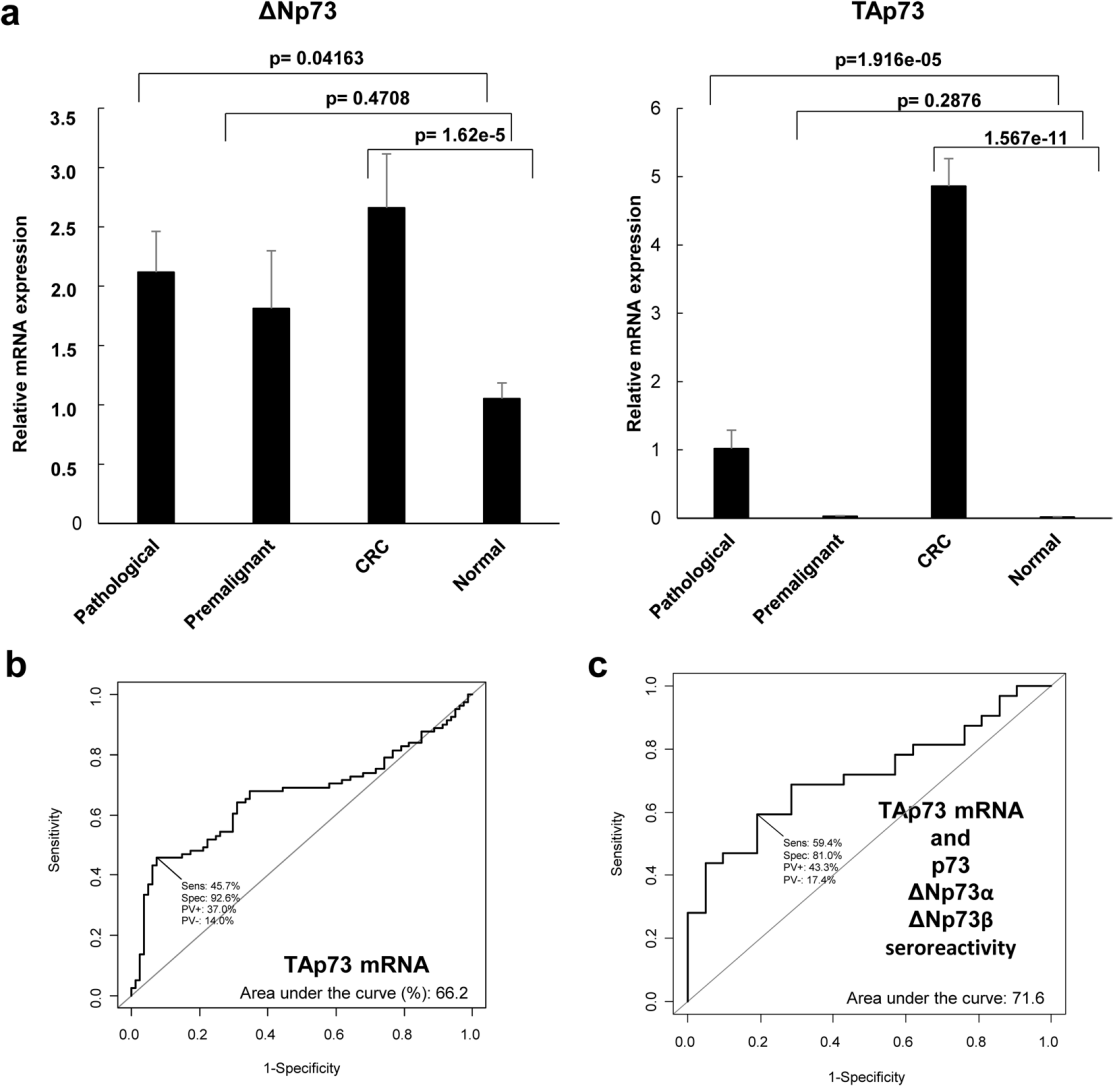
Evaluation of ΔNp73 and TAp73 relative mRNA expression levels in paired normal and pathological tissue samples by qPCR. (**a**) For ΔNp73, relative mRNA expression 2.12 ± 0.35 (mean ± SEM) was found in pathological tissue, while values of 1.05 ± 0.13 (mean ± SEM) were found in normal tissue *(p-*value = 0.04163). For TAp73, relative mRNA expression of 1.02 ± 0.27 (mean ± SEM) was found in pathological tissue, while values of 0.017 ± 0.002 (mean ± SEM) were found in normal tissue (*p-*value 1.916e-5). Trimmed mean was used in all cases to compensate for extreme values. All measurements were taken in duplicate. (**b**) Assessment of the predictive value of the relative mRNA expression levels for TAp73 and ΔNp73 in tissue samples of healthy individuals *vs* premalignant and CRC subjects was performed through ROC curves, with only TAp73 a showing significant predictive value. (**c**) Evaluation of the predictive value of the combination of the seroreactivity to p73, ΔNp73α, or ΔNp73β, and the mRNA expression levels of TAp73 showed an overall AUC of 71.6%.

Next, since ΔNp73 overexpression is a common hallmark in pathological tissue of colorectal cancer patients, we aimed to evaluate whether the differences in the seroreactivity to ΔNp73 could be explained by the overexpression of ΔNp73 in tissue. To address this question, a qPCR analysis of ΔNp73 and TAp73 mRNA expression levels from paired pathological/normal samples from premalignant, and CRC patients, together with controls (Fig. 4a), whose plasma samples were previously analyzed by ELISA-based test, was done.

A mean value of 1.05 ± 0.13 relative mRNA expression levels for ΔNp73 was found in the normal tissue, whereas in the pathological group a mean value of 2.12 ± 0.35 was found. On the other hand, for TAp73, mean relative mRNA expression values of 0.017 ± 0.002 and 1.02 ± 0.27 were found for the normal and pathological tissue, respectively. In addition, relative mRNA expression levels were 0.03 ± 0.005, and 4.86 ± 0.40 in the premalignant lesions and in the CRC tumor tissue, respectively. Moreover, although higher values were observed for ΔNp73 in the premalignant lesions comparing controls, results were only statistically significant either for ΔNp73 or TAp73 comparing CRC *vs* controls and the pathological group *vs* controls. In addition, no correlation was observed between the relative mRNA expression levels of ∆Np73 or TAp73 isoforms and the specific seroreactivity to either ∆Np73 or p73.

According to the significance of the group comparisons, we next evaluated the usefulness of the relative mRNA expression levels to discriminate between the pathological and the control group by ROC curves (Figs 4b and S4), to finally determine if the humoral immune response to p73 and its isoforms together with the relative mRNA expression levels of TAp73 and/or ∆Np73 would improve the diagnostic effectiveness observed for the seroreactivity to p73, and ΔNp73α, and ΔNp73β. In the cohort of colorectal cancer and colorectal cancer premalignant patients used in this study, only TAp73 showed individually a relevant AUC (66.2%). In combination, TAp73 mRNA expression levels and the seroreactivity to p73, and ΔNp73α, and ΔNp73β did not improve the performance of the seroreactivity to detect CRC and premalignant individuals, showing an AUC of 71.6% and a specificity and sensitivity of 81.0% and 59.4% (Fig. 4c).

Collectively, our data suggest that only the deregulation of ∆Np73 seems to be enough to produce a specific seroreactivity to ΔNp73α, and ΔNp73β either in premalignant individuals or CRC patients, which shows an important diagnostic effectiveness to discriminate between colorectal cancer patients, colorectal premalignant individuals and controls using plasma of patients.

### Evaluation of the selective seroreactivity to the ΔNp73α and ΔNp73β isoforms

**Figure 5:**
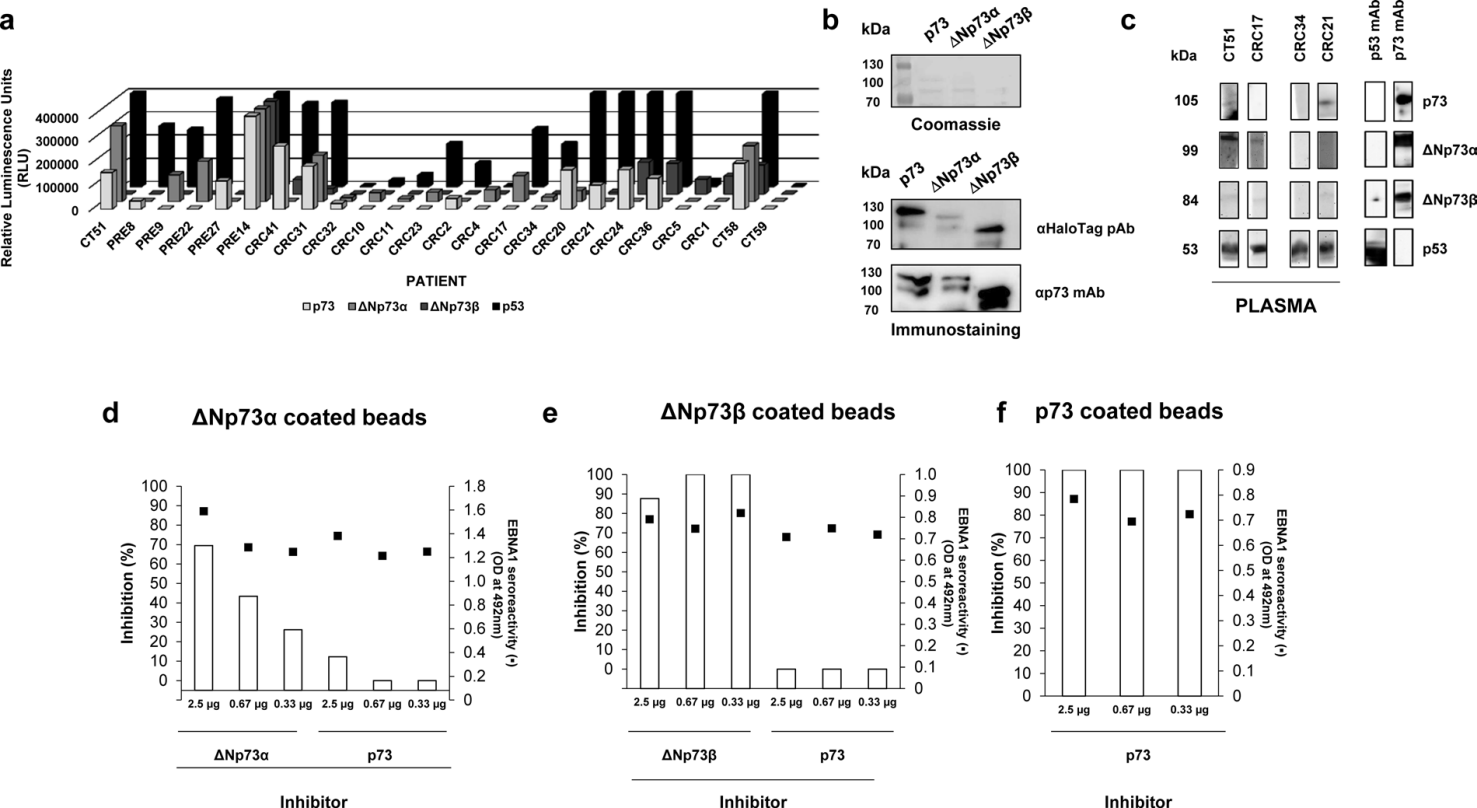
Verification of the selective seroreactivity to ΔNp73 isoforms. (**a**) The evaluation of the specific seroreactivity to the different proteoforms highlight that 22 individuals showed a specific seroreactivity to any of the proteoforms. CT58 as control of reactivity to all proteins and CT59 as control of no reactivity to any protein were also represented. (**b**) To verify this selective seroreactivity by WB, proteins were expressed fused to the HaloTag and 6xHis-tag at the C-terminal. Coomassie blue staining and immunostaining using antibodies directed to the expressed proteins or their Halo- and 6xHis-tags were used to verify protein expression. In cropped images, expressed proteins were run in the same gel under same experimental conditions and processed in parallel. (**c**) WB using indicated plasma samples was carried out to verify the differential seroreactivity observed by ELISA. Purified proteins were run on a single-well gel and transferred into a nitrocellulose membrane. Bands corresponding to integral proteins were cut in same-size bands and incubated with indicated individual plasma samples or primary antibodies. Inhibition assays (**d–f**) showed a specific seroreactivity to unique epitopes present in ∆N proteoforms. Pooled plasma samples seroreactive to (**d**) ΔNp73α or (**e**) ΔNp73β were preincubated with different amounts of indicated proteoforms as inhibitors prior to seroreactive analysis. (**f**) Seroreactivity of a pooled plasma sample seroreactive to p73 inhibited with increasing amounts of p73 was used as control of the assay. EBNA1 seroreactivity was determined as internal control of the experiment since more than 95% of human possessed antibodies against this protein of the capsid of the Epstein-Barr virus. All experiments were performed in duplicate.

Since the autoantibody levels to ΔNp73α showed a higher diagnostic ability than the reference canonical protein, and due to the differential seroreactivity observed among the isoforms, we further investigated whether the differential seroreactivity to the isoforms could be associated to the presence of unique epitopes present in the ΔNp73 isoforms (Fig. 5a). To this end, the proteins were also produced fused to HaloTag to analyze by WB the specific seroreactivity to the isoforms avoiding any tag-bias in the analysis, and to use the proteins in competitive inhibition assays that further assessed this specific seroreactivty.

First, protein purification through Ni-NTA columns was verified by Coomassie Blue staining and WB with an α-HaloTag pAb that recognizes the C-terminal tag on every antigen and a α-p73 monoclonal antibody that specifically recognizes the presence of p73 and its isoforms fused to HaloTag, respectively (Fig. 5b). The purified fusion proteins were transferred to nitrocellulose membranes to probe them with plasma samples selectively reactive to p73, ΔNp73α, or ΔNp73β by luminescence (Fig. 5c), using p53 as control. Interestingly, the differential seroreactivity was also detected by WB, where indicated premalignant individuals or colorectal cancer patients showed at similar extents the differential seroreactivity found by luminescence ELISA-based tests.

To finally confirm the specific seroreactivity to the ΔNp73 isoforms different from that of the canonical p73 protein, inhibition experiments were performed using the purified proteins, the IVTT expression of p73 and its isoforms fused to GST, and plasma from patients seroreactive in a specific manner to the different isoforms. We observed a dose-dependent inhibition of the autoantibody signal specific to ΔNp73α, and ΔNp73β, not able to be abrogated by the canonical p73 protein (Fig. 5d,e). On the other hand, the signal of the same plasma samples preincubated with the indicated purified proteins remained constant in their recognition to EBNA1. Finally, as control and to avoid any concern about the specificity of the assay, the autoantibody signal to p73 was found to be fully inhibited using the canonical p73 protein as inhibitor (Fig. 5f). Collectively, these data confirm the presence in ΔNp73 isoforms of unique specific epitopes not present in p73.

## Discussion

Among the vast number of different cancer circulating biomarkers, altered proteins during tumor formation and progression that are able to induce a humoral response in cancer patients provide an effective, reliable, and noninvasive tool for cancer screening and preclinical diagnosis^31–35^. Although the molecular mechanisms and the subsequent production of autoantibodies against self-proteins of the humoral response in cancer are far from being known, it is reported that proteins present alterations, including alternative splicings, punctual mutations, truncations, overactivation, aberrant glycosylations, overexpression, and/or aberrant degradation^31,32^.

Novel high-throughput proteomic approaches have accelerated the identification of these circulating serum autoantibodies and their respective target proteins as potential cancer biomarkers. One of the main advantages of this approach for cancer diagnosis is that antibodies are highly stable molecules with a long tradition in immunoassays, facilitating their standardization in serological assays. In the last years, the development of serologic screening of natural and/or recombinant protein microarrays has led to the discovery of hundreds of novel proteins target of autoantibodies in cancer patients^16,33–41^. As a discovery platform, these technologies hold the promise to complete the cancer autoantibody immunome once microarrays containing the total proteome become available (considering one canonical protein per gene)^34,42,43^. However, proteins carrying important functions in the cells are usually tightly regulated and suffer from an extensive regulation at transcriptional level, and thus they usually codify for multiple isoforms. Accordingly, by using the canonical sequences of proteins, autoantigens produced as a consequence of alternative splicings that could play a key role for cancer diagnosis might be lost as autoantibody targets.

Here, we have analyzed the potential different seroreactivity of proteoforms codified by alternative splicing of the p73 member of the p53-family. The p53-family composed of p53, p63 and p73, which regulates many vital biological processes, including cell differentiation, proliferation, and cell death/apoptosis, is well-known to be tightly controlled at transcriptional level^44–47^. Indeed, about 40 different proteoforms have been theoretically described for p73 and at least 20 and 10 for p53 and p63, respectively, due to alternative splicing. Although not all of them have been experimentally confirmed, it is surprising that none of the proteoforms different from that of the canonical sequences have been tested as target of autoantibodies in cancer patients. To our knowledge, the only study where several isoforms of the p53-family have been analyzed was related to oral lichen planus, a chronic inflammatory disease of oral mucosa^18,20^. In that study, the authors analyzed the contribution of the linear epitopes of several isoforms of p53, p63 and p73 by WB, and found that two patients reacted against the p63 isoforms tested, with p63β showing the strongest reactivity, and one of these patients reacted also to p73; showing these patients the worst lesions.

Importantly, the cancer humoral immune response is a polyclonal response, which recognizes linear and/or conformational immunogenic epitopes within the same protein^33–35,48,49^. In addition, recent advances in native protein display technologies suggest that conformational-dependent discontinuous epitopes may represent up to 90% of the total B-cell response and are often dependent on the secondary or tertiary structure of proteins^50^. Accordingly, the best alternative to be used for the seroreactivity analysis of p53-family proteoforms should be those immunoassays were the proteins are correctly folded at secondary and tertiary level. Here, we have used a methodology well-known to be useful for the analysis of either conformational or linear immunogenic epitopes, where the proteins are correctly oriented in the assay. More importantly, the proteins are expressed in a *mammalian milieu* and *in situ* during the assay, and their folding is equivalent to that observed in cancer cells, avoiding at the same time any degradation or precipitation issue during their purification or storage. This methodology derived from the NAPPA and RAPID NAPPA methodologies, with minor modifications, allowed to survey for the analysis of the seroreactivity with the integral and correctly folded proteins^15,26,51^.

In this context, we hypothesized that the isoforms of the p53-family might produce a differential seroreactivity in cancer patients since they possess important differences in their primary sequences, affecting their 3D folding. Thus, these proteoforms should contain both unique linear and conformational epitopes in their structure, which might be target of autoantibodies in cancer patients being useful for cancer diagnosis and patient monitoring. As a proof of concept of our hypothesis, we focused on the member of the p53 family codifying theoretically for more potential isoforms: p73; and specifically in two cancer-associated proteoforms: ΔNp73α and ΔNp73β that are specifically overexpressed in colorectal cancer^47,52^, among other cancers. In addition, these isoforms differed in the primary sequence of the protein and consequently in their 3D folding and in the surface electrostatic potential compared to the canonical p73 protein as depicted in Fig. 1. In general, the N- and C-terminal ends are the most immunogenic regions. However, cancer patients do not elicit a humoral immune response to the N-terminal end of p73^19^, as evaluated by WB. Therefore, as observed by modeling the p73 proteoforms, both the N- and C-terminal ends of ΔNp73α and ΔNp73β are located at the protein surface leading to important changes in the 3D folding and at electrostatic level, which at least for ΔNp73α and ΔNp73β might produce an immunodominance not observed in p73.

As the most important goals of the study, we unequivocally demonstrated that ∆Np73 proteoforms produced a specific seroreactivity in colorectal cancer patients and premalignant individuals different from that observed for the p73 canonical protein. We also observed that the N-terminal end of ΔNp73 is an important target of autoantibodies in contrast to p73, since several patients showed a specific reactivity against ΔNp73α, which possesses a different N-terminal end than that of p73. In addition, we showed that the seroreactive diagnostic ability of ∆Np73α is higher than that of either p73 or the ∆Np73β proteoform, and more importantly, this specific seroreactivity significantly improved the diagnostic effectiveness of p73 to discriminate colorectal cancer patients or premalignant individuals from controls. Furthermore, the autoantibody levels were higher in premalignant individuals than in colorectal cancer patients, suggesting that the diagnostic ability of the specific seroreactivity to the ΔNp73α proteoform might be associated to early cancer detection. This was confirmed by ROC curve analyses, showing that ΔNp73α possess a higher diagnostic ability than either p73 or ΔNp73β for discriminating premalignant individuals from controls. In addition, by measuring CEA in the same samples, we have found that ΔNp73 seroreactivity is more sensitive and more specific than CEA to predict premalignant lesions. Since the identification of ΔNp73 seroreactivity is cheap and easy to assess in clinics, this finding might have an important impact due to the fact that the seropositivity to ΔNp73α proteoform would justify intervention to prevent development of CRC.

Collectively, we propose an evaluation of the different proteoforms of the p53-family to elucidate which proteoforms should be included in diagnostic panels for cancer beyond the canonical sequences of p53, p63 and p73. Moreover, in this study we have increased the complexity of the cancer humoral immune response to the p53-family in cancer patients, suggesting that the proteoforms derived from the alternative splicings of p53 and p63 might also possess a higher diagnostic ability than the canonical proteins, as occurs with ∆Np73α in comparison to canonical p73. Therefore, an evaluation of the seroreactivity of the multiple proteoforms of the p53-family should be particularly valuable to determine whether the cancer diagnostic ability of the different proteoforms might be associated to specific cancers or might improve the diagnostic ability of the canonical proteins for cancer and early cancer diagnosis. On the other hand, the analysis of independent patient cohorts, larger number of samples and samples of different disease, including samples from irritable bowel syndrome and other cancers, would be of great interest to establish their actual clinical utility for the early diagnosis of colorectal cancer and the detection of individuals carrying premalignant lesions. In this sense, further research is guaranteed to try to determine whether ∆Np73 seroreactivity would reflect chronic inflammation (like CEA or CA19-9) or whether it is more specific of colorectal premalignant lesions than that of CRC as our results suggest, and more importantly to assess the actual clinical management utility of ∆Np73 seroreactivity by determining its association with clinic-pathological parameters, which might help identifying patients with higher risk of cancer progression and also assist in selecting the most efficient personalized treatments.

In summary, this is the first study to demonstrate the utility of the specific ∆Np73α autoantibodies different from that of the canonical p73 to discriminate colorectal cancer patients and premalignant patients from healthy controls. Remarkably, ∆Np73α seroreactivity showed a higher diagnostic ability than that of the canonical protein, and in combination both proteoforms were able to improve their diagnostic ability alone either for detection of CRC patients or premalignant individuals from controls. Further validation with a larger cohort of samples and comprehensive analysis of the multiple different proteoforms of the p53-family is warranted.

## Methods

### Patient plasma

The Institutional Ethical Review Boards of the Complutense University of Madrid (UCM), ISCIII, Hospital Clínico San Carlos (Madrid), and La Paz Hospital (Madrid) approved this study on biomarker discovery (CEI PI 45). Plasma samples were obtained from the Hospital Clínico San Carlos, Hospital La Paz and IMDEA-FOOD (Genyal platform) after approval of the Ethical Review Boards of these institutions. All subjects in the study gave their written informed consent to participate and all experiments were performed in accordance with relevant guidelines and regulations.

For the analysis of the presence of specific autoantibodies to ΔNp73 isoforms different from that of p73 autoantibodies in plasma samples of cancer patients, a panel of 145 plasma samples from colorectal cancer patients and premalignant subjects (low- or high-grade adenomas in the colon), and healthy control individuals (healthy individuals, and FOBT positive and colonoscopy negative individuals) were used (Table 1, and Supplementary Table S1). Plasma samples were collected using a standardized sample collection protocol and stored at −80 °C until use^35,37,38,53^.

Tissue samples from the same individuals were histopathologically confirmed and obtained from the Biobanks of the IdISSC and IdIPAZ, which belongs to the National Biobank Net (ISCIII) cofounded with FEDER funds, of the same Hospitals between June 2015 and March 2016 prior to initiating any treatment.

### *In silico* modeling of the proteins

Structural models in PDB format were generated using the Phyre^2^ program^54^. Final 3D-structure models were obtained with PyMOL (Schrödinger, LCC, New York) and the electrostatic surface potential for the proteins was predicted by the Adaptive Poisson Boltzmann Solver (APBS) program^55^.

### Gateway plasmid construction, gene cloning, DNA preparation and protein expression

Sequence-verified full-length cDNA plasmid containing *TP53*, or *TP73* in flexible pDONR221 vector system was obtained from the publicly available DNASU Plasmid Repository (https://dnasu.org/DNASU/)^56^. Alternatively, ΔNp73α and ΔNp73β genes were PCR amplified with specific oligonucleotides and the pcDNA3.1 containing the full-length ΔNp73α or ΔNp73β cDNAs. PCR products were directly cloned in pDONR221 by a BP clonase reaction (Invitrogen) according to manufacturer instructions^14,51^. The ORFs were transferred by LR clonase reactions (Invitrogen, Carlsbad, CA), alternatively, to a pANT7_cGST vector for *in vitro* protein expression, or a pANT7-cHaloHis vector, developed in the laboratory, for bacterial protein expression^15^ to get p73 and ΔNp73 isoforms expressed as fusion proteins to GST or HaloTag in the C-terminal end, respectively^51^. All donor and expression plasmids were sequence verified prior to a subsequent use.

To obtain high-quality supercoiled DNA, plasmids were transformed into TOP10 *E. coli* cells and grown in 250 mL Luria Bertani (LB) supplemented with the adequate antibiotic (100 μg/mL for Ampicilin and 40 μg/mL for kanamycin). Plasmid DNA was purified using the NucleoBond^®^ Xtra Midi kit (Macherey-Nagel Inc., Bethlehem, PA). Proteins were expressed using T7 reticulocyte lysate (Promega Corporation, Madison, WI) per manufacturer’s recommendations to carry out the ELISA studies. To validate the specific seroreactivity to ΔNp73, proteins were also expressed in bacteria. Briefly, BL21 (DE3) *E. coli* cells were grown in 250 mL of LB until an OD of 0.6 was reached, and, then, expression was induced with 0.4 mM IPTG for 48 h at 16 °C and 220 rpm. Then, the bacteria were centrifuged for 10 min at 4000 rpm and the pellet resuspended in 5 ml 50 mM phosphate and 300 mM NaCl containing 1%-lauroyl-sarcosinate and maintained in ice for 30 min, and further lysed with 1% Triton-X100 in ice for 20 min before three subsequent cycles of sonication for 30 s. Then, the supernatant was clarified by centrifugation and proteins purified using Ni-NTA resin (CliniSciences) per manufacturer’s instructions^57^.

### SDS-PAGE and western blot analysis

SDS-PAGE and western blot analysis to assess protein quality and specific protein seroreactivity in selected individuals’ plasma were performed as previously reported^57^. Briefly, 2 µl of the *in vitro* transcription/translation (IVTT) protein extracts or 500 ng of purified p73, ΔNp73α, and ΔNp73β proteins were run in 10% SDS-PAGE and transferred to nitrocellulose membranes (Hybond-C extra). After blocking, membranes were incubated overnight at 4 °C with optimized dilutions of specific monoclonal antibodies against p73, GST tag, HaloTag, or indicated plasma samples. Immunodetection on the membranes was achieved by using HRP-conjugated secondary antibodies (Supplementary Table S2). The chemiluminescence signal was developed with ECL Western Blotting Substrate (Thermo Scientific) and detected on a Fujifilm LAS-3000 Imager (Fujifilm).

### ELISA-based tests

The IVTT product was diluted 1:10 in PBS and transferred to overnight coated Maxisorp plates (Nunc) with 50 µL anti-GST pAb (GE Healthcare) diluted 1:100 in PBS. After washing, plates were blocked in blocker casein solution (Pierce) diluted 1:1 in PBS for 2 h, and then incubated for 1 h at room temperature with plasma samples at indicated dilutions. After washing, 50 µL of anti-human IgG antibody-HRP conjugated (Jackson) diluted at indicated dilutions in PBS, Tween 20 0.1% (PBST) and 3% skimmed milk was added per well. Alternatively, GST tagged-proteins were detected with anti-GST (Cell Signaling), or anti-p73 (Pierce) monoclonal antibodies followed by the incubation with the HRP conjugated-anti-mouse IgG (Sigma Aldrich, Missouri, MO) diluted 1:3000. The signal was finally developed with 50 µL per well of SuperSignal ELISA Femto Max Sensitivity (Pierce, Rockford, IL) and chemiluminescence recorded on a FLUOstar Optima (BMG LABTECH, Ortenberg, Germany). All seroreactive values against p73, ΔNp73α, and ΔNp73β of the samples tested in the study are shown in Supplementary Table S1.

CEA concentration (ng/ml) in plasma samples was determined using a specific immunoassay test kit (Sigma Aldrich), following the manufacturer’s recommendations. All CEA values of the samples tested in the study are shown in Supplementary Table S1.

### Inhibition assay of the specific autoantibody seroreactivity

To avoid any tag-bias concern and to verify the specific selective seroreactivity of some of the CRC patients to p73 and its isoforms, a competitive inhibition assay of the autoantibody binding was developed. Briefly, 0.5 µl of HaloTag magnetic beads were equilibrated following the manufacturer’s instructions and incubated overnight at 4 °C and constant shaking with 0.1 µg of purified integral p73, ΔNp73α, and ΔNp73β proteins fused to HaloTag and 6xHisTag for their covalent immobilization. After covalent immobilization of the proteins to the magnetic beads, beads were extensively washed with PBST containing 0.05% Triton-X100, and finally with 0.1 M glycine, pH 2.7 to remove any unspecific protein bound to beads. After elution, the beads coupled with the proteins were blocked with 3%-BSA-PBST, and subsequently incubated with pooled plasma of patients seroreactive to either ΔNp73α or to ΔNp73β diluted 1:200 preincubated with indicated amounts of proteins for 2 h at room temperature, using seroreactivity to p73 and EBNA1 as controls of the assay.

### qPCR analysis of ∆Np73 and TAp73

Quantitative real-time polymerase chain reaction was performed in a Light Cycler apparatus (Roche Diagnostics) using the LightCycler-FastStart DNA Master SYBR Green I Kit (Roche Diagnostics). The primer sets for ΔNP73, and the reaction conditions were as described previously^58,59^. The housekeeping gene succinate dehydrogenase complex subunit A (SDHA) was used to normalize gene expression results.

### Statistical analysis

All statistical analyses were done with Microsoft Office Excel and the R program. For the analysis of ELISA and qPCR datasets, data distribution using the Shapiro-Wilk test and variance homogeneity using the Bartlett test was first evaluated. Since data normality and homogeneous variances were discarded in all cases, we assessed whether the means of control individuals, and premalignant individuals and CRC groups were statistically different from each other using the non-parametric U-Mann Whitney test. All given values correspond to the trimmed mean ± SEM to compensate for extreme values. *p-*values < 0.05 were considered statistically significant. Individual autoantibody against each indicated target was evaluated as marker in plasma of premalignant individuals, CRC patients and control individuals by a ROC curve. ROC curves were constructed with the R program (version 3.2.3) using the R package Epi^60^; and the corresponding AUC and the maximized sensitivity and specificity values were calculated.

## Supplementary information

Supplementary Information

## Data Availability

All data generated or analysed during this study are included in this published article (and its Supplementary Information Files).
