# Supplementary material for: The specific seroreactivity to ∆Np73 isoforms shows higher diagnostic ability in colorectal cancer patients than the canonical p73 protein
Source: Sci Rep. 2019 Sep 19;9:13547. doi: 10.1038/s41598-019-49960-x (PMC6753153; doi:10.1038/s41598-019-49960-x)
Supplement: Supplementary file 1 — Supplementary Information [file 41598_2019_49960_MOESM1_ESM.pdf]

The specific seroreactivity to ΔNp73 isoforms shows higher diagnostic ability in colorectal cancer patients than the canonical p73 protein

María Garranzo-Asensio[1,2], Ana Guzmán-Aránguez[1], Carmen Povés[3], María Jesús Fernández-Aceñero[4], Ana Montero-Calle[2], María Ángeles Ceron[4], Servando Fernandez-Diez[3], Nuria Rodríguez[5], Marta Gómez de Cedrón[6], Ana Ramírez de Molina[6], Gemma Domínguez[*,7], Rodrigo Barderas[*,2]

[1]Departamento de Bioquímica y Biología Molecular, Facultad de Óptica y Optometría, Universidad Complutense de Madrid, E-28040 Madrid, Spain, [2]UFIEC, Chronic Disease Programme, Instituto de Salud Carlos III, Majadahonda E-28220, Madrid, Spain, [3]Gastroenterology Unit, Hospital Universitario Clínico San Carlos, E-28040 Madrid, Spain, [4]Surgical Pathology Department, Hospital Universitario Clínico San Carlos, E-28040 Madrid, Spain, [5]Medical Oncology Department, Hospital Universitario La Paz, E-28046 Madrid, Spain, [6]Molecular Oncology and Nutritional Genomics of Cancer, IMDEA-FOOD, E-28049, Madrid, Spain, [7]Departamento de Medicina, Facultad de Medicina, Instituto de Investigaciones Biomédicas "Alberto Sols", CSIC-UAM, E-28029 Madrid, Spain

*Co-senior authors. To whom correspondence should be addressed:

Supplemental Table S1. Individual clinical information of the colorectal cancer patients and controls used in the study.

Plasma code	Clasification	Group	Gender	Age	CRC stage (TNM)	p73 (RLU)*	ΔNp73α (RLU)*	ΔNp73β (RLU)*	CEA (ng/ml)
CT1	Asymptomatic	Control	M	51		4192	0	0	0.00
CT2	Asymptomatic	Control	M	56		0	0	2222	0.00
CT3	Asymptomatic	Control	F	51		0	0	0	0.00
CT4	Asymptomatic	Control	F	38		381	0	310	0.00
CT5	Asymptomatic	Control	M	42		1347	2488	553	0.00
CT6	Asymptomatic	Control	F	46		2224	4124	3589	0.00
CT7	Asymptomatic	Control	F	45		2676	4871	7488	0.00
CT8	Asymptomatic	Control	M	41		2697	3330	2361	0.00
CT9	Asymptomatic	Control	M	41		0	0	743	0.00
CT10	Asymptomatic	Control	F	51		678	0	0	0.00
CT11	Asymptomatic	Control	M	49		0	0	0	0.00
CT12	Asymptomatic	Control	F	47		0	0	0	0.00
CT13	Asymptomatic	Control	M	38		0	0	3031	0.00
CT14	Asymptomatic	Control	F	57		4189	0	0	0.00
CT15	Asymptomatic	Control	F	34		0	0	0	0.00
CT16	Asymptomatic	Control	M	34		0	0	0	0.00
CT17	Asymptomatic	Control	F	55		0	0	0	0.00
CT18	Asymptomatic	Control	F	46		1245	0	691	0.00
CT19	Asymptomatic	Control	M	40		0	0	3278	0.00
CT20	Asymptomatic	Control	F	50		0	0	1434	3.67
CT21	Asymptomatic	Control	F	41		0	0	3340	0.00
CT22	Asymptomatic	Control	F	37		0	0	3279	0.00
CT23	Asymptomatic	Control	F	48		0	0	7485	0.00
CT24	Asymptomatic	Control	F	52		0	1376	0	0.00
CT25	Asymptomatic	Control	M	49		0	0	9720	0.00
CT26	Asymptomatic	Control	F	38		0	0	4467	0.00
CT27	Asymptomatic	Control	M	31		0	0	6814	0.00
CT28	Asymptomatic	Control	F	47		300	1964	4379	0.00
CT29	Asymptomatic	Control	F	51		0	4274	7072	0.00
CT30	Asymptomatic	Control	F	45		0	287	2205	0.00
CT31	Asymptomatic	Control	F	34		0	278	1054	0.00
CT32	Asymptomatic	Control	F	50		0	0	46	0.00
CT33	Asymptomatic	Control	F	51		0	12457	0	0.00
CT34	Asymptomatic	Control	F	38		0	0	0	0.00
CT35	Asymptomatic	Control	F	44		12853	4143	0	0.00
CT36	Asymptomatic	Control	F	46		38056	12665	4050	0.00
CT37	Asymptomatic	Control	F	49		0	0	0	0.00
CT38	Asymptomatic	Control	F	52		0	0	0	0.00
CT39	Asymptomatic	Control	F	61		0	0	0	0.00
CT40	Asymptomatic	Control	M	39		0	0	0	2.77
CT41	Asymptomatic	Control	F	29		49439	14076	0	0.00
CT42	Negative colonoscopy	Control	F	46		0	0	0	0.00
CT43	Negative colonoscopy	Control	F	40		0	0	0	0.00
CT44	Negative colonoscopy	Control	F	43		0	0	0	0.00
CT45	Negative colonoscopy	Control	M	35		0	0	0	0.00
CT46	Negative colonoscopy	Control	F	48		0	0	0	0.00
CT47	Negative colonoscopy	Control	M	41		0	0	0	0.00
CT48	Negative colonoscopy	Control	M	36		0	0	0	0.00
CT49	Negative colonoscopy	Control	F	41		0	0	0	0.00
CT50	Negative colonoscopy	Control	M	67		1168000	1078000	0	0.00
CT51	Negative colonoscopy	Control	F	61		157000	326000	0	0.00
CT52	Negative colonoscopy	Control	F	42		0	0	0	0.00
CT53	Negative colonoscopy	Control	F	57		244000	0	106000	0.00
CT54	Negative colonoscopy	Control	F	56		472000	145000	0	2.81
CT55	Negative colonoscopy	Control	M	57		0	0	0	0.00
CT56	Negative colonoscopy	Control	M	58		0	0	0	0.00
CT57	Negative colonoscopy	Control	F	52		261000	309000	187000	0.00
CT58	Negative colonoscopy	Control	M	64		278000	262000	207000	0.00
CT59	Negative colonoscopy	Control	M	70		0	0	0	0.00
CT60	Negative colonoscopy	Control	F	57		0	0	0	0.00
CT61	Negative colonoscopy	Control	F	55		0	0	0	0.00
CT62	Negative colonoscopy	Control	M	57		0	0	0	0.00
CT63	Negative colonoscopy	Control	F	52		0	0	0	0.00
CT64	Negative colonoscopy	Control	F	49		0	0	0	0.00
CT65	Negative colonoscopy	Control	F	64		0	0	0	0.00
CT66	Negative colonoscopy	Control	F	61		285977	188716	167767	0.00
CT67	Negative colonoscopy	Control	F	53		277703	220001	185480	0.00
CT68	Negative colonoscopy	Control	F	59		368277	229277	239277	0.00
PRE1	Adenoma	Premalignant group/pathological	M	60		1070000	887000	508000	0.00
PRE2	Adenoma	Premalignant group/pathological	M	59		1612000	1054000	1200000	0.10
PRE3	Adenoma	Premalignant group/pathological	F	57		389000	480000	493000	0.00
PRE4	Adenoma	Premalignant group/pathological	F	64		326000	164000	308000	0.45
PRE5	Adenoma	Premalignant group/pathological	F	54		301000	926000	548000	0.00
PRE6	Adenoma	Premalignant group/pathological	F	61		0	0	0	3.14
PRE7	Adenoma	Premalignant group/pathological	M	65		0	0	0	0.00
PRE8	Adenoma	Premalignant group/pathological	M	60		34000	0	0	0.00
PRE9	Adenoma	Premalignant group/pathological	M	59		0	115000	0	4.09
PRE10	Adenoma	Premalignant group/pathological	M	59		497000	389000	0	0.00
PRE11	Adenoma	Premalignant group/pathological	M	57		1090000	830000	582000	6.76
PRE12	Adenoma	Premalignant group/pathological	M	40		367845	254845	183326	0.00
PRE13	Adenoma	Premalignant group/pathological	F	50		673000	471000	479000	2.35

PRE14	Adenoma	Premalignant group/pathological	M	67		857000	691000	499000	0.00
PRE15	Adenoma	Premalignant group/pathological	M	62		540000	467000	380000	0.00
PRE16	Adenoma	Premalignant group/pathological	F	60		0	0	0	0.00
PRE17	Adenoma	Premalignant group/pathological	F	75		365000	405000	241000	0.00
PRE18	Adenoma	Premalignant group/pathological	M	70		187000	222000	110000	0.00
PRE19	Adenoma	Premalignant group/pathological	M	61		150000	116000	40000	0.00
PRE20	Adenoma	Premalignant group/pathological	F	59		197000	241000	125000	0.00
PRE21	Adenoma	Premalignant group/pathological	M	70		0	0	0	0.00
PRE22	Adenoma	Premalignant group/pathological	F	60		0	174000	0	3.43
PRE23	Adenoma	Premalignant group/pathological	F	59		278000	116000	0	2.85
PRE24	Adenoma	Premalignant group/pathological	F	54		0	0	0	0.47
PRE25	Adenoma	Premalignant group/pathological	F	67		0	0	0	0.00
PRE26	Adenoma	Premalignant group/pathological	M	56		0	0	0	0.00
PRE27	Adenoma	Premalignant group/pathological	M	61		121339	0	0	0.00
PRE28	Adenoma	Premalignant group/pathological	F	58		0	0	0	0.00
PRE29	Adenoma	Premalignant group/pathological	F	67		0	0	0	0.00
PRE30	Adenoma	Premalignant group/pathological	F	60		322455	171455	234455	0.09
PRE31	Adenoma	Premalignant group/pathological	M	40		367845	254845	183326	0.00
PRE32	Adenoma	Premalignant group/pathological	F	68		337686	245410	196473	0.00
PRE33	Adenoma	Premalignant group/pathological	F	64		169029	133444	146760	0.00
PRE34	Adenoma	Premalignant group/pathological	F	59		281450	130002	129287	0.00
CRC1	CRC	CRC group/pathological	M	66	I	0	0	77000	5.50
CRC2	CRC	CRC group/pathological	M	75	I	45276	0	0	3.00
CRC3	CRC	CRC group/pathological	M	89	I	0	0	0	1.30
CRC4	CRC	CRC group/pathological	M	86	II	0	50308	0	117.30
CRC5	CRC	CRC group/pathological	F	60	II	0	0	63000	0.80
CRC6	CRC	CRC group/pathological	M	75	IIA	92955	354955	258955	0.00
CRC7	CRC	CRC group/pathological	M	75	IIA	0	0	0	0.00
CRC8	CRC	CRC group/pathological	M	75	IIA	0	0	0	0.70
CRC9	CRC	CRC group/pathological	F	75	IIA	77332	40529	15407	37.10
CRC10	CRC	CRC group/pathological	F	82	IIA	0	38000	0	8.40
CRC11	CRC	CRC group/pathological	F	82	IIA	0	10930	0	8.40
CRC12	CRC	CRC group/pathological	F	82	IIA	0	0	0	8.40
CRC13	CRC	CRC group/pathological	F	77	IIA	359000	32000	0	50.00
CRC14	CRC	CRC group/pathological	F	59	IIA	89100	62756	6795	0.00
CRC15	CRC	CRC group/pathological	M	87	III	110111	80483	115031	9.84
CRC16	CRC	CRC group/pathological	M	85	III	0	0	0	4.00
CRC17	CRC	CRC group/pathological	M	63	IIIA	0	111664	0	231.00
CRC18	CRC	CRC group/pathological	M	78	IIIA	0	0	0	0.00
CRC19	CRC	CRC group/pathological	F	66	IIIA	0	0	0	1.80
CRC20	CRC	CRC group/pathological	F	60	IIIA	168966	46746	0	6.30
CRC21	CRC	CRC group/pathological	M	71	IIIA	102685	0	8196	0.00
CRC22	CRC	CRC group/pathological	M	61	IIIB	149608	137608	60608	1.48
CRC23	CRC	CRC group/pathological	F	85	IIIC	0	40085	0	0.00
CRC24	CRC	CRC group/pathological	M	75	IIIC	169320	0	137466	1.20
CRC25	CRC	CRC group/pathological	M	77	IIIB	0	0	0	3.20
CRC26	CRC	CRC group/pathological	F	66	IV	109487	96821	50662	19.90
CRC27	CRC	CRC group/pathological	F	51	IV	46346	58753	33823	13.90
CRC28	CRC	CRC group/pathological	F	66	IV	109463	53709	50340	1539.00
CRC29	CRC	CRC group/pathological	M	84	IV	236071	31707	65202	7.20
CRC30	CRC	CRC group/pathological	M	72	IV	0	0	0	45.30
CRC31	CRC	CRC group/pathological	F	58	IV	184000	199000	23000	5.70
CRC32	CRC	CRC group/pathological	F	87	IV	24000	16000	0	4.00
CRC33	CRC	CRC group/pathological	F	74	IV	0	0	0	8.90
CRC34	CRC	CRC group/pathological	F	59	IV	0	18000	0	0.00
CRC35	CRC	CRC group/pathological	F	55	IV	19278	12888	47169	0.00
CRC36	CRC	CRC group/pathological	F	74	IV	132755	0	132517	6.96
CRC37	CRC	CRC group/pathological	M	82	IV	0	0	0	316.00
CRC38	CRC	CRC group/pathological	M	84	IV	32583	2774	91248	4.80
CRC39	CRC	CRC group/pathological	F	58	IV	84957	3247	27593	0.50
CRC40	CRC	CRC group/pathological	M	54	IV	19687	35489	17085	2.40
CRC41	CRC	CRC group/pathological	M	54	IV	271000	0	62000	2.40
CRC42	CRC	CRC group/pathological	F	51	IV	57217	45816	10953	0.50
CRC43	CRC	CRC group/pathological	F	77	IV	0	0	0	2.40

*RLU, Relative Luminescence Units

Supplementary Table S2. Information of the primary and secondary antibodies or reagents used in the study.

Primary Antibodies						
**Antibody target**	**Clonality**	**Source**	**Application**	**Dilution**	**Provider**	**Reference**
p73	Monoclonal	Mouse	WB	1:1000	Pierce	38c674.2
GST	Monoclonal	Mouse	WB	1:5000	Cell Signaling	2624
GST	Polyclonal	Rabbit	ELISA	1:100	GE Healthcare	27457701

HRP-labeled secondary antibodies or HRP-labeled reagents					
**Name**	**Clonality**	**Source**	**Application**	**Dilution**	**Provider**
HRP anti-mouse	Polyclonal	Goat	WB	1:2500	Dako
HRP anti-rabbit	Polyclonal	Goat	WB	1:3000	BioRad
HRP Human-IgG-biotin	Polyclonal	Goat	ELISA	1:20000	Jackson
HRP-streptavidin			ELISA	1:1000	Raybiotech
HRP Human-IgG	Polyclonal	Goat	ELISA	1:3000	Dako

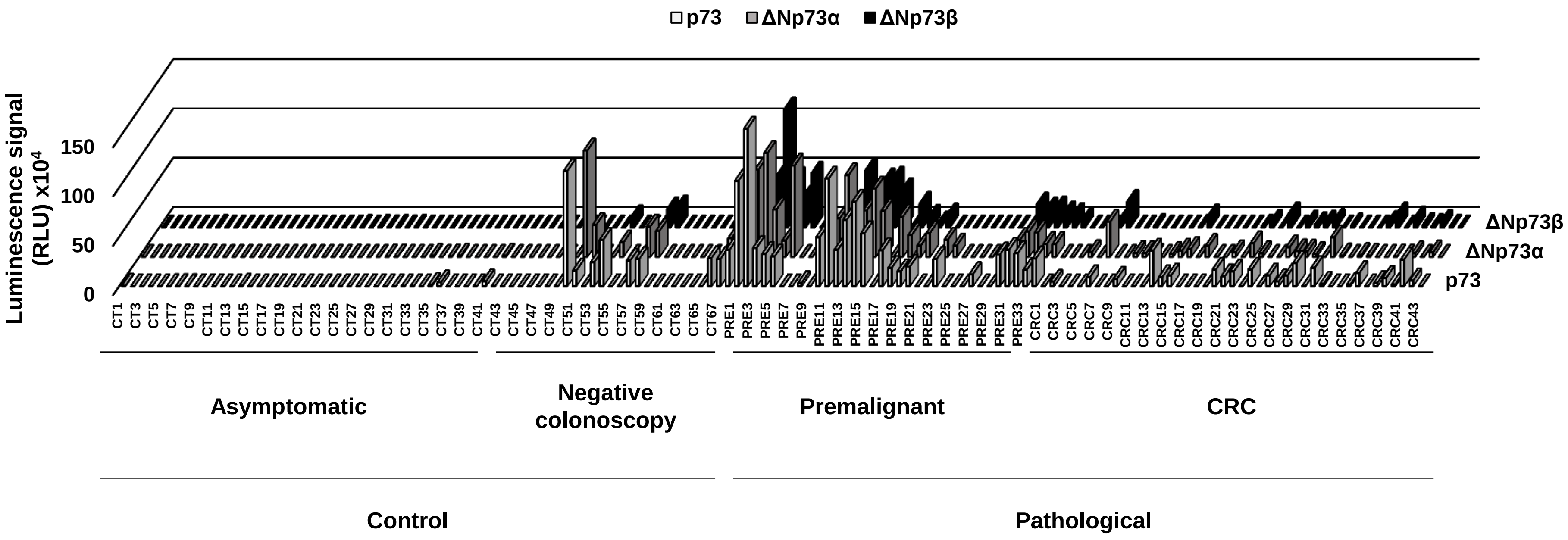

Supplementary Fig. S1.

Evaluation of the seroreactivity to p73, ΔNp73α, and ΔNp73β. Plasma from colorectal cancer patients, and premalignant individuals showed specific reactivity against p73, ΔNp73α and ΔNp73β in comparison to control samples (asymptomatic and control individuals with negative colonoscopy). Plasma was tested by the ELISA-based Luminescence test using the proteins expressed in vitro fused to GST in the C-terminal end. Results are represented as bar graph comparing the seroreactivity of each plasma sample against all analyzed proteins. Remarkably, since during the course of the investigations we found six seroreactive controls with negative colonoscopy that were diagnosed cancer lesions non-associated to CRC (not included in the study and in the statistical analysis), those seroreactive control individuals with negative colonoscopy depicted in the figure are subjected to extensive clinical management for further assessment.

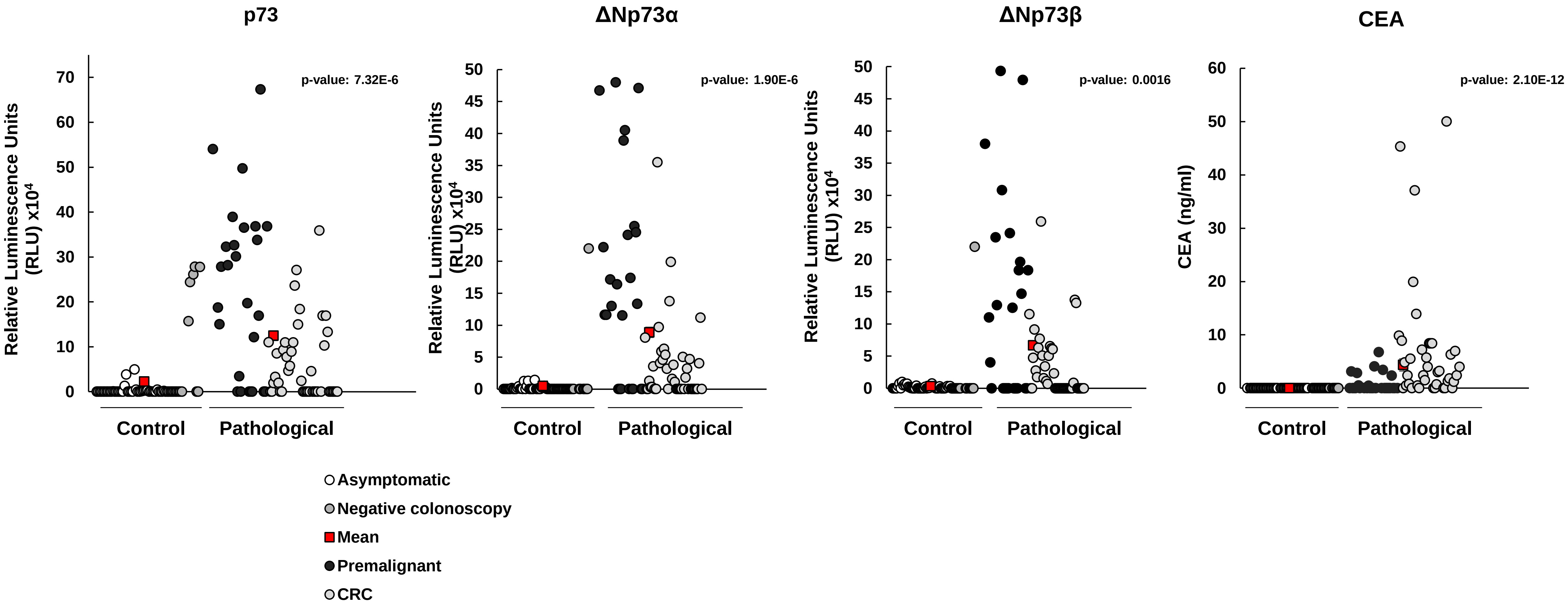

Supplementary Fig. S2.

Dot-plot analysis of the seroreactivity to p73, ΔNp73α, and ΔNp73β in comparison to CEA values. The individual seroreactivity of each patient to p73, ΔNp73α, and ΔNp73β with the characteristics of all plasma samples is shown. Statistical differences (p-value=7.32e-06, 1.90e-06, and 0.0016) were found comparing the mean value of the luminescence signal obtained for each proteoform (p73, ΔNp73α, and ΔNp73β, respectively) for the control group and the pathological groups. Statistical differences (p-value=2e-12) were also found for CEA values in discriminating control and pathological groups.

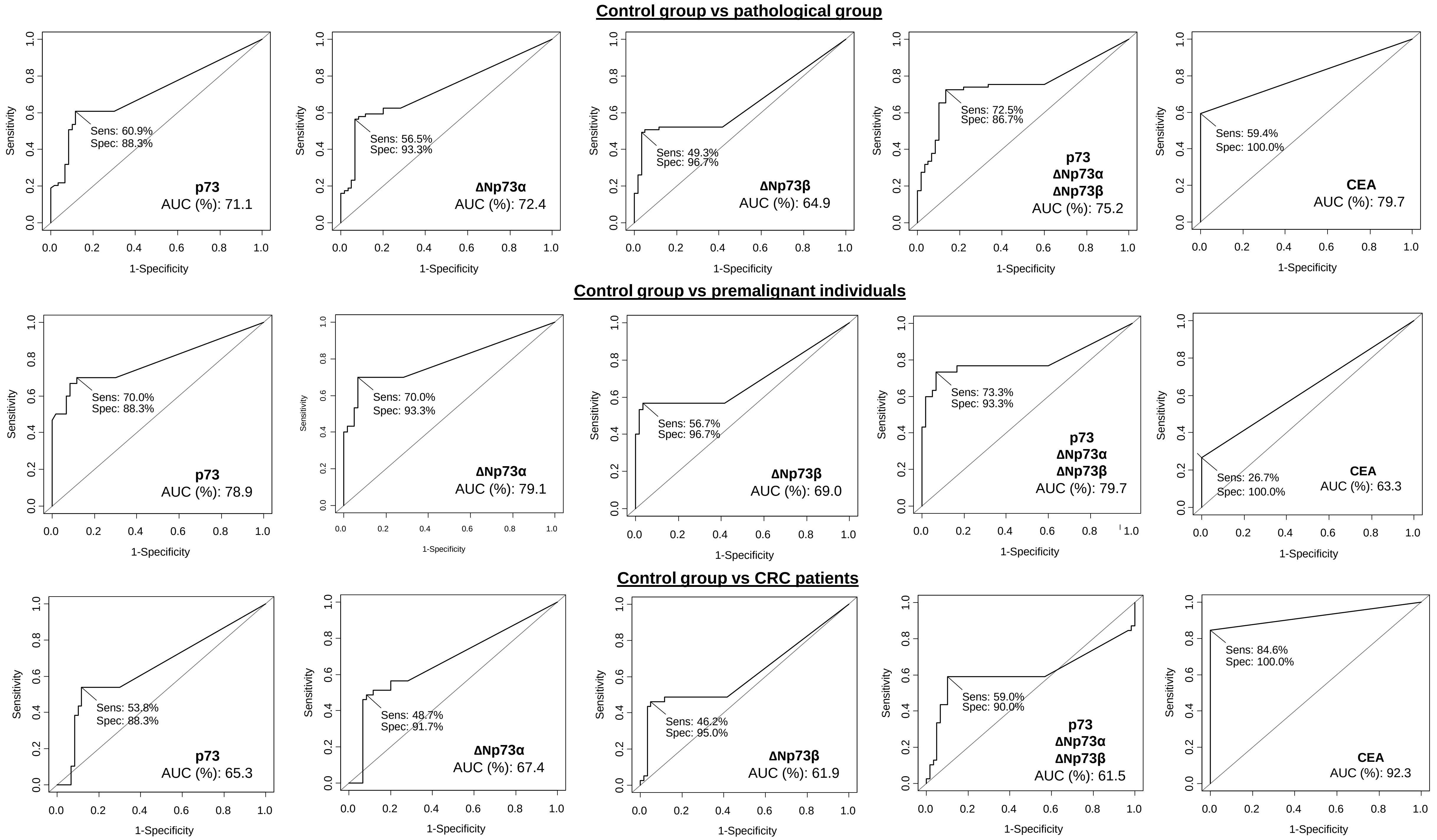

Supplementary Fig. S3.

Individual and combined ROC curves for the seroreactivity to p73, ΔNp73α, or ΔNp73β were calculated in comparison to CEA values to assess their predictive value according to the indicated comparisons.

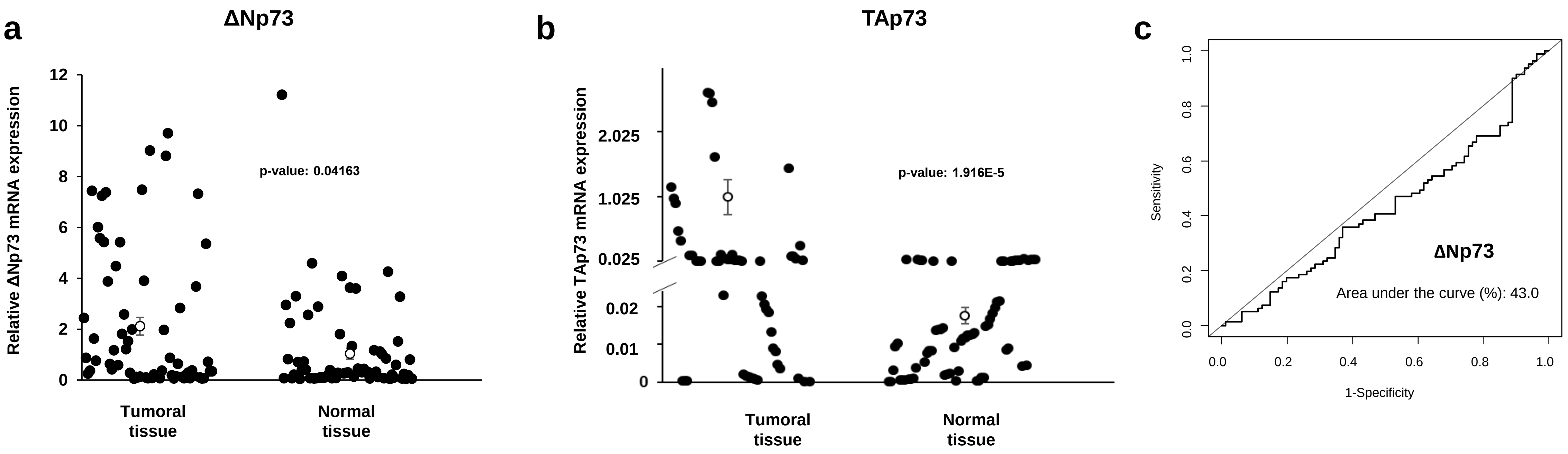

Supplementary Fig. S4.

Dot-plot representation of the evaluation of ΔNp73 and TAp73 relative mRNA expression levels in paired normal and pathological tissue samples by qPCR. (a) For ΔNp73, relative mRNA expression 2.12±0.35 (mean±SEM) was found in pathological tissue, while values of 1.05±0.13 (mean±SEM) were found in normal tissue (p-value=0.03642). (b) For TAp73, relative mRNA expression of 1.02±0.27 (mean±SEM) was found in pathological tissue, while values of 0.017±0.002 (mean±SEM) were found in normal tissue (p-value=2.662e-5). (c) Assessment of the predictive value of the relative mRNA expression levels for ΔNp73 in tissue samples of healthy individuals and premalignant and CRC subjects was performed through ROC curves.

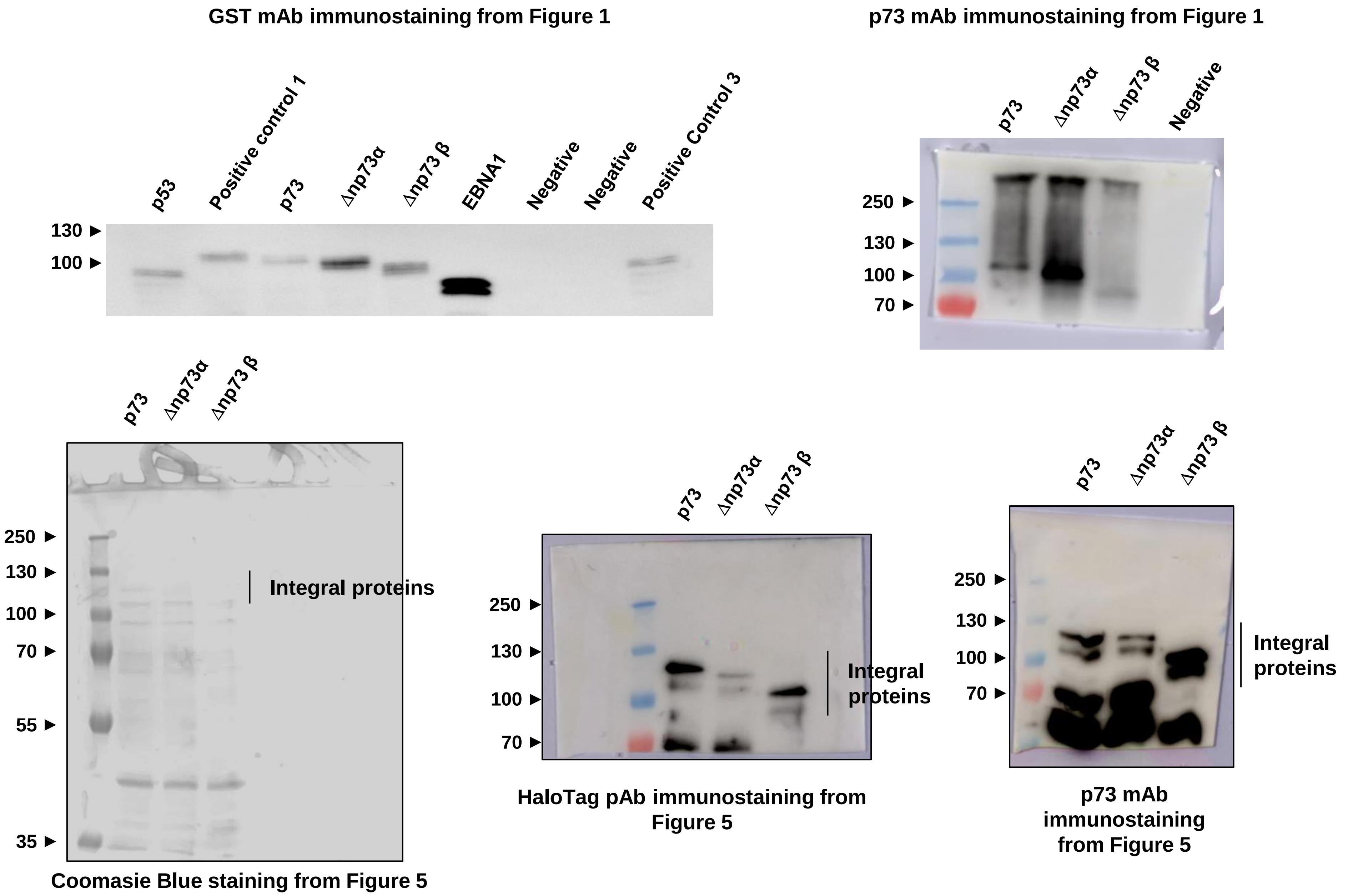

Supplementary Fig. S5.
Full-length blots/gels from figures 1 and 5 are presented.
